# Ethnomedicinal plant knowledge and practice of the Oromo ethnic group in southwestern Ethiopia

**DOI:** 10.1186/1746-4269-4-11

**Published:** 2008-04-29

**Authors:** Haile Yineger, Delenasaw Yewhalaw, Demel Teketay

**Affiliations:** 1 Department of Biology, Jimma University, P.O. Box 5195, Jimma, Ethiopia; 2 FSC Africa, 4 Asoyi Road, East Legon, UPO LPMB 11, Legon, Accra, Ghana

## Abstract

An ethnomedicinal study was conducted to document the indigenous medicinal plant knowledge and use by traditional healers in southwestern Ethiopia from December 2005 to November 2006. Data were collected from 45 randomly selected traditional healers using semi-structured interviews and observations. Sixty-seven ethnomedicinal plant species used by traditional healers to manage 51 different human ailments were identified and documented. Healers' indigenous knowledge was positively correlated with their reported age but not with their educational level. High degree of consensus was observed among traditional healers in treating tumor (locally known as *Tanacha*), rabies (*Dhukuba Seree*) and insect bite (*Hadhaa*). The use of more than one species was significantly cited for remedy preparations. The reported abundance of the ethnomedicinal plant species varied significantly with respect to the presence of multiple uses of the reported species. Our results showed that ethnomedicinal plant species used by healers are under serious threat due to several factors, which indicates the need for urgent attention towards their conservation and sustainable utilization.

## Background

Ethnomedicinal plants have been used since ancient time for human healthcare and still remain the most widely used medication system in developing and least developed nations like Ethiopia where over 80% of the population is dependent on traditional medicines [1]. The reliance of people on ethnomedicine has been for reasons of cost-effectiveness, acceptability, biomedical benefits and accessibility. There has been a continuous growth of demand for herbal medicines globally [2]. The demand has been increasing as a result of growth of human populations and the frequently inadequate provision of modern medicine [3]. Numerous species of ethnomedicinal plants are threatened in most of developing nations mainly due to overexploitation, overgrazing, habitat loss and alteration, destructive harvesting techniques, unsustainable trade and deforestation [4]. The loss of medicinal plant species has also been aggravated by the erosion of the age old accumulated indigenous knowledge on traditional use and management of these plants as its transfer system is widely known to be poor [1,5,6].

In Ethiopia, ethnomedicinal plant knowledge and use is underreported and most of the studies made so far are not focused on specific ethnic group or agro ecological zone of the country. Therefore, the main objective of this study was to document the ethnomedicinal plant species used to manage human ailments and the associated indigenous knowledge in and around Gilgel Gibe Hydropower Reservoir, southwestern Ethiopia.

## Materials and methods

### Study area and population

**Figure 1:**
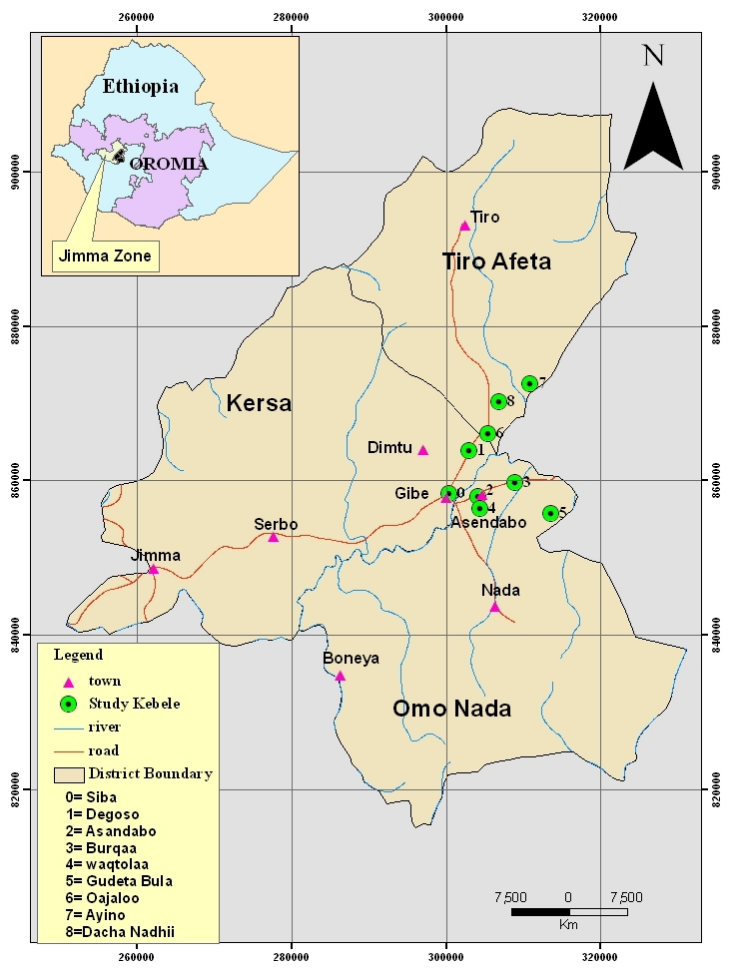
Location of the study *Kebeles *in the three selected districts of Jimma Zone, Ethiopia.

The study was carried out in nine *Kebeles *(the smallest administrative units in Ethiopia) belonging to three districts (Omo Nada, Kersa and Tiro Afeta) around Gilgel Gibe Hydropower Reservoir, Jimma Zone, southwestern Ethiopia. Jimma Zone is one of the administrative zones in Oromia Region of Ethiopia. It is one of the major coffee growing areas of the country contributing much to the economy of the nation. The population of Omo Nada, Kersa and Tiro Afeta is 254417, 329629 and 130554, respectively [7]. The study *Kebeles *under the aforementioned districts were Siba, Degoso, Asandabo, Burqaa, Waqtolaa, Gudeta Bula, Qajaloo, Ayino and Dacha Nadhii (Figure 1). The study area is located at about 265 km southwest of the capital Addis Ababa and 65 km Northeast of Jimma town at 07°42'37" – 07°53'50" N and 037°11'22" – 037°20'36" E. It has an altitudinal range of 1675 to 2094 m, a mean annual temperature of 19.2°C and receives an annual rainfall ranging from 1200 – 2800 mm. Evergreen montane thickets and shrubs are typical vegetation types of the area. Cultivating crops (maize, teff, sorghum, barley, pulses and false banana) and rearing of livestock are the major socioeconomic activities of the local people.

The people of the study area belong to the Oromo ethnic group, which is the largest ethnic group in Ethiopia [8], consisting about 40% of the population of the nation [9]. The people of Oromo speak *Afaan *Oromo, which is one of the Kushitic language families of the Afro-Asiatic language group [10]. The Oromo people living in different parts of the country were engaged in different socio-economic activities as pastoralists and sedentary agriculturalists for centuries. Currently, the dominant socioeconomic activity of the Oromo people is mixed farming. The major religions of the Oromos are now Islam and Christianity [11]. The people have a long tradition of social organization, the *Gada *System, by which they maintain their social, political and cultural systems. Moreover, traditional healers of the Oromo people are well known in treating many illnesses with medicines made from local medicinal plant species and individuals also were known to use plants for home remedies for minor illnesses.

### Data collection

Ethnobotanical data were collected from December 2005 to November 2006. Ethical clearance was obtained from Jimma University Ethics and Review Committee and written consent to undertake the study was sought from district leaders of Omo Nada, Kersa and Tiro Afeta. The full names and residential addresses of traditional healers residing in the nine selected *Kebeles *within 20 Km distance from Gilgel Gibe Dam were exhaustively identified and registered with the help of local administrators, local people, translators and field assistants. Individuals who were indicated to know and practice at least one medicinal plant species were considered as traditional healers in this study. A total of 90 healers were identified and registered. From this list, 45 healers were selected randomly and considered as the study subjects. Semi-structured interviews were then employed and observations [12] made to collect ethnomedicinal data with the help of local language translators and field assistants. Some of the events during data collection are shown in Additional file 1.

Data on age, sex, level of education, occupation, religion, ethnicity, human diseases treated, local names of plants used, degree of management (wild/cultivated), abundance, parts used, condition of plant part used (fresh/dried), methods of remedy preparations, remedy preservation (storage), dosage prescriptions, routes of remedy administration, noticeable adverse effects of remedies, use of antidotes for adverse effects, indigenous knowledge transfer, other uses of the ethnomedicinal plant species, existing threats to these species and traditional conservation practices were gathered during the interviews. The authors also made observations in the field on the general habitats and the ethnomedicinal plants collected by accompanying traditional healers, translators and field assistants. The collected voucher specimens were pressed, numbered, dried, identified and deposited at Jimma University Regional Herbarium and The National Herbarium (ETH) in Addis Ababa University. Identification of specimens was made with the help of herbarium materials, experts and taxonomic keys in the Flora of Ethiopia and Eritrea [13-20]. The local names of diseases are included in the following text within brackets.

### Data analyses

Facilities in MS Excel spread sheet were utilized to make simple calculations, determine proportions and draw bar graphs. Informant consensus factor (ICF) values were determined following [21] to evaluate the consensus among traditional healers. These values were calculated as: ICF = nuc – ns/nuc – 1, where nuc = number of use citations, ns = number of species used for each use citation. Moreover, the level of fidelity (Fl) was computed to determine the most important species used by the healers according to [22] as: Fl (%) = (SF/TF)100, where SF = frequency of citation of a species for a specific ailment and TF = total number of citations of that species.

Chi-square (X^2^), Spearman Rank Correlation and Binomial Tests were also employed to analyze ethnomedicinal data using SPSS 12.0.1 software package. Chi-square test was used to determine if there was a significant difference (*p *< 0.05) on i) the mean number of medicinal plant species reported by each healer versus district; and ii) the abundance of medicinal plant species with respect to plant part used, condition of plant part used (fresh/dried), marketability, and added values of the medicinal species.

The Spearman Rank Correlation Test was employed to evaluate whether there was significant (*p *< 0.05) correlation between i) the diversity of medicinal plant species recorded and altitude; ii) the age of traditional healers and the number of ethnomedicinal plant species reported; and iii) the educational level of healers and the number of species reported.

The Binomial Test was used to evaluate whether i) the indigenous knowledge was transferred to generations; ii) modernization had any influence on the transfer of the indigenous knowledge; iii) taboos were present during collection and processing of remedies, iv) mixtures of species were used more frequently, v) healers were preserving (storing) remedies, vi) dosage prescriptions were similar for different age groups, vii) remedies were devoid of adverse effects after administration, viii) healers were using antidotes for noticeable adverse effects, ix) the reported species were mainly marketable, x) the species reported were threatened in the study area, xi) the medicinal species had added values; and xii) most healers were practicing conservation of the reported species.

## Results

### Particulars of traditional healers

All of the traditional healers involved in the study were male, married, Muslims and farmers, except one, who was a self-employed traditional medicine practitioner. The traditional healers belonged to the Oromo ethnic group. Their reported ages ranged from 25 to 87, and each traditional healer had a mean family number of eight. The majority (53%) were illiterate and those who could read and write constituted 33% while 13% attended grades one to four.

### Reported human ailments and consensus of healers

Fifty one different human ailments were treated by the traditional healers using various ethnomedicinal plant species. High degree of consensus (ICF = 0.50) was observed among the traditional healers in treating tumor (*Tanacha*) and that this disease was treated by employing *Tapinanthus globiferus *(A. Rich.) Tieghem, *Gloriosa superba *L. and *Plumbago zeylanica *L. (Table 1).

**Table 1:** Healers' consensus factor and fidelity levels

**Ailment**	**ICF**	**Species**	**Fidelity level (%)**
Tumor	0.50	*Gloriosa superba *L.	66.67
		*Plumbago zeylanica *L.	40.00
Rabies	0.33	*Ricinus communis *L.	100.00
		*Salix subserrata *Willd.	50.00
		*Afrocarpus falcatus *(Thunb.) C. N. Page	50.00
Insect bite	0.33	*Alysicarpus quartinianus *A. Rich.	33.33
		*Cassia arereh *Del.	50.00
Rheumatism	0.29	*Croton macrostachyus *Del.	42.86
		*Plumbago zeylanica *L.	20.00
		*Justicia schimperiana *(Hochst. ex Nees) T. Anders.	50.00
		*Momordica foetida *Schumach.	33.33
		*Calpurnia aurea *(Ait.) Benth.	25.00
		*Clausena anisata *(Willd.) Benth.	33.33
*Rissaa*	0.25	*Croton macrostachyus *Del.	14.29
		*Cyathula uncinulata *(Shrad.) Schinz	50.00
		*Clerodendrum myricoides *(Hochst.) Vatke	40.00

Rabies (*Dhukuba Seree*) and insect bite (*Hadhaa*) shared the second highest degree of consensus (ICF = 0.33) (Table 1). Freshly pounded and squeezed leaves of *Ricinus communis *L. were reported to be used along with milk in treating patients of rabies (*Dhukuba Seree*). Crushed leaves of *Salix subserrata *Willd. and *Afrocarpus falcatus *(Thunb.) C. N. Page were also used in fresh form, mixed with water and milk, to treat the same disease.

On the other hand, traditional healers treat insect bite (*Hadhaa*) by applying leaf poultices of *Alysicarpus quartinianus *A. Rich. and *Canavalia africana *Dunn. Leaves or roots of *A. quartinianus *were also crushed while fresh and bandaged over infected sites with a clean piece of cotton cloth. The stem bark of *Cassia arereh *Del. was also pounded either in fresh or dried conditions and dressed on infected sites with a piece of cotton cloth to treat this ailment.

The consensus factor among traditional healers for rheumatism was 0.29. Eleven ethnomedicinal plant species were effective to treat this ailment: *Dregea schimperi *(Decne.) Bullock, *Croton macrostachyus *Del., *P. zeylanica*, *Justicia schimperiana *(Hochst. ex Nees) T. Anders., *Clutia abyssinica *Jaub. & Spach., *Celtis africana *Burm. *f*., *Momordica foetida *Schumach., *Ocimum gratissimum *L., *Calpurnia aurea *(Ait.) Benth., *Bersama abyssinica *Fresen. and *Clausena anisata *(Willd.) Benth.

*Rissaa *was still another ailment reported with considerable consensus among the traditional healers with an ICF value of 0.25. Leaves of *C. macrostachyus *and *Cyathula uncinulata *(Shrad.) Schinz were crushed in fresh, mixed with water and very small amount of it (only base of a cup) was reported to be drunk to treat this ailment. The roots of *Clerodendrum myricoides *(Hochst.) Vatke were also reported to be crushed in fresh, squeezed and the juice mixed with milk and eventually administered orally in very small amount while leaves of *Desmodium repandum *(Vahl) DC. were crushed in fresh and pasted over the body.

### Indigenous knowledge and diversity of medicinal plant species

The correlation between the age of traditional healers and the number of medicinal species reported by each healer was highly significant. The statistical details are presented in Table 2. Older traditional healers mentioned more number of medicinal plant species than younger healers. Cross tabulation of the result on the mean number of species reported by each traditional healer versus district also showed highly significant difference. However, no significant correlation was observed between the educational level of traditional healers and the number of species reported by each healer.

**Table 2:** Statistical tests of significance

**Type of test**	**Variables tested**	**r**	**χ^2^**	**df**	**p-value**
Chi-square	Mean no spp Vs District		286.401*	27	0.0001
	Spp abundance Vs Parts used		232.134*	76	0.0001
	Spp abundance Vs Condition used (Fresh/Dried)		167.170*	12	0.0001
	Spp abundance Vs Marketability		167.538*	8	0.0001
	Spp abundance Vs Added values		17.343*	4	0.0002
Spearman Rank Correlation	Diversity of spp Vs Altitude	-0.290**			0.0001
	Age of healers Vs No spp	0.446**			0.0001
	Educational level of healers Vs No spp	0.055*			0.483

* Significant at 0.05 level (two tailed); ** Correlation is significant at the 0.01 level (two tailed).

High diversity of medicinal plant species was recorded with a total of 67 species, 65 genera and 35 botanical families (see Additional file 2). Highly significant negative correlation was observed between altitude where medicinal plants were collected and the number of medicinal plant species recorded. Fabaceae was represented with the highest number of species (10). This was followed by Euphorbiaceae (6 species), Asteraceae (5 species), Lamiaceae (4 species) and Solanaceae (3 species). Acanthaceae, Asclepiadaceae, Cucurbitaceae, Loranthaceae, Malvaceae, Myrsinaceae, Ranunculaceae, Rubiaceae and Rutaceae were represented with two species each while the other 21 families were represented with one species each (Table 3). The majority (76%) of these medicinal plant species were wild while few (13%) were both wild and cultivated and the remaining (12%) were cultivated.

**Table 3:** Medicinal plant families in the study area with the corresponding numbers of species

**Families**	**Species**	**Proportion (%)**
Fabaceae	10	14.93
Euphorbiaceae	6	8.96
Asteraceae	5	7.46
Lamiaceae	4	5.97
Solanaceae	3	4.48
Rutaceae	2	2.99
Rubiaceae	2	2.99
Ranunculaceae	2	2.99
Myrsinaceae	2	2.99
Malvaceae	2	2.99
Loranthaceae	2	2.99
Cucurbitaceae	2	2.99
Asclepiadaceae	2	2.99
Acanthaceae	2	2.99
Vitaceae	1	1.49
Ulmaceae	1	1.49
Salicaceae	1	1.49
Podocarpaceae	1	1.49
Poaceae	1	1.49
Plumbaginaceae	1	1.49
Phytolaccaceae	1	1.49
Oxalidaceae	1	1.49
Oleaceae	1	1.49
Myricaceae	1	1.49
Moraceae	1	1.49
Menispermaceae	1	1.49
Melianthaceae	1	1.49
Hypericaceae	1	1.49
Flacourtiaceae	1	1.49
Dioscoriaceae	1	1.49
Colchicaceae	1	1.49
Boraginaceae	1	1.49
Apocynaceae	1	1.49
Amaranthaceae	1	1.49
Verbenaceae	1	1.49

**Figure 2:**
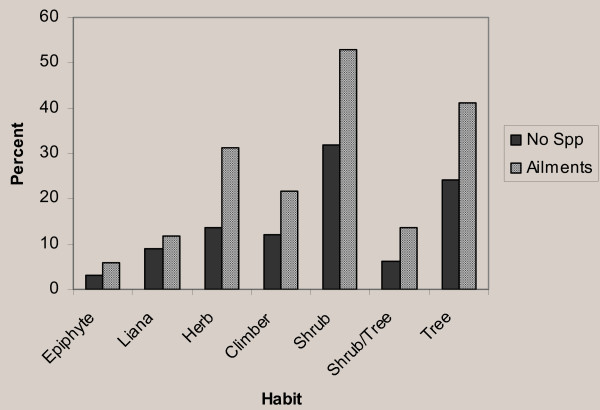
Medicinal plant habits and proportion of human ailments treated.

Woody species (21 shrub species, 32% and 16 tree species, 24%) were more frequently used for traditional medicine preparations than other life forms. Herbs, climbers and lianas accounted for 14%, 12%, and 9%, respectively (Figure 2).

According to traditional healers, extensive indigenous plant use knowledge was retained and transferred orally to a selected family member. Most of the traditional healers reported that modernization had no effect on the transfer of the indigenous knowledge to generations. The majority of traditional healers also reported that there were no taboos associated with medicinal plant collection and uses in the study area.

### Plant parts used, methods of preparation and application

**Figure 3:**
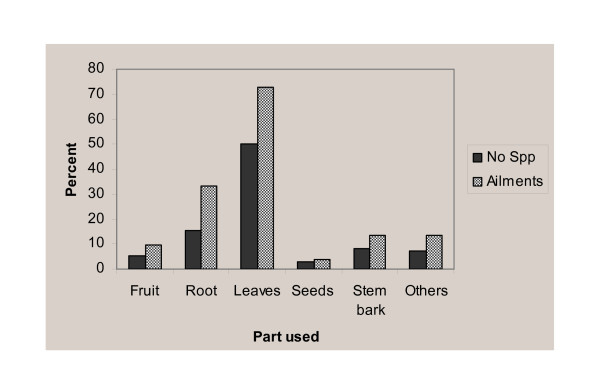
Medicinal plant parts used to the treatment of human ailments.

Leaves (48 species, 50%), roots (15 species, 16%) and stem bark (8 species, 8%) were the most cited plant parts for remedy preparations. Considerable numbers of species were also sought for their fruits (5 species, 5%) (Figure 3). The various plant parts were mostly (56 species, 72%) processed fresh while some (16 species, 21%) were used dried and others (6 species, 8%) either fresh or dried.

Medicinal plant parts were reported mostly to be crushed (35%), squeezed (27%) and powdered (12%) during preparation of remedies (Table 4). Mixtures of different species were used to treat most of the reported ailments than the use of a single species. About 65%, 41% and 27% of the reported ailments were treated with remedies prepared by crushing, squeezing and powdering, respectively. Most of the traditional healers were not preserving or storing traditional medicinal preparations for use at another time.

**Table 4:** Reported methods of preparation of traditional medicine

**Methods of preparation**	**Frequency**	**Proportion (%)**
Crushed	60	35.09
Squeezed	46	26.90
Powdered	20	11.70
Pounded	13	7.60
Concocted	12	7.02
Extracted with cold water	11	6.43
Decocted	4	2.34
Warmed	2	1.17
Smoked	1	0.58
Extracted by boiling stem	1	0.58
Enclosed in a piece of close	1	0.58

**Figure 4:**
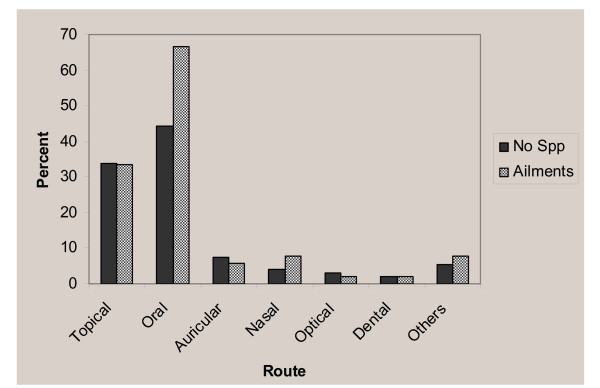
Administration routes of traditional medicine.

The administration of remedial preparations were mainly oral (42 species, 44%) and on top of the body (32 species, 34%) (Figure 4). According to healers, preparations were prescribed to patients differently for different age groups. The dosage prescription for children was mostly lower than for adults. Dosages were estimated using lids, spoons, cups, glasses, pinches or handfuls. The amounts of remedy and prescription rates were generally dependent on the degree and duration of the ailment. Treatment durations varied between 1 and 7 days.

Traditional healers also indicated that their remedies were devoid of any adverse effects. However, some mild adverse effects like abdominal pain, diarrhoea, inflammation, vomiting, unconsciousness and high rate of breathing were reported for some of the remedial preparations. Almost all of the informants were not using antidotes for noticeable adverse effects of traditional medicines applied.

### Threats to medicinal plants and their conservation

The ethnomedicinal plant species were mainly reported as rare (36 species, 40%) and abundant (27 species, 30%) while others as very abundant (17 species, 19%) and very rare (10 species, 11%). Highly significant difference was observed between the plant parts used for medicinal purposes and the abundance of the medicinal species in the study area. The use of plant remedy in fresh or dried conditions also showed highly significant variation on the abundance of the medicinal plant species. Non-marketable ethnomedicinal plants were more frequently reported. The abundance of medicinal species also varied significantly with regard to the marketability of the medicinal plant species.

**Figure 5:**
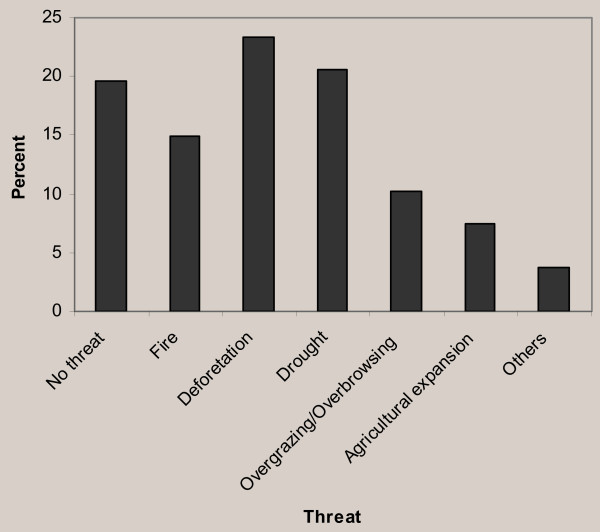
Threats to medicinal plant species of human importance.

The number of ethnomedicinal plant species threatened in the study area was significantly higher. The most cited threats to ethnomedicinal plants of the area were deforestation (25 species, 23%), drought (22 species, 20.56%), fire (16 species, 15%), overgrazing/over browsing (11 species, 10%) and agricultural expansion (8 species, 7%). Twenty one species (20%) were reported to have no apparent threat (Figure 5). Moreover, the absence of practice by traditional healers to conserve or recuperate ethnomedicinal plants of the area was highly significant.

**Figure 6:**
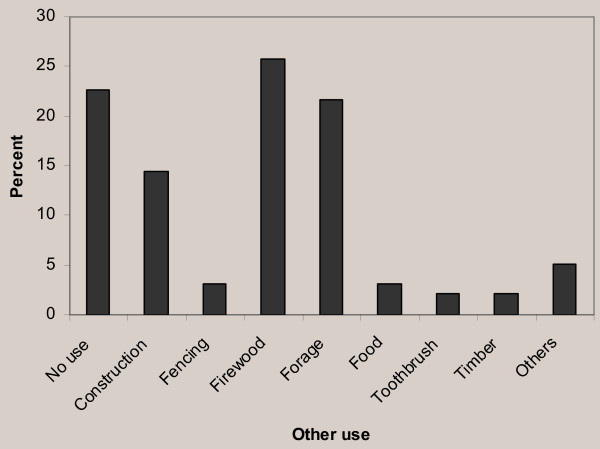
Other uses of medicinal plant species.

Ethnomedicinal plant species having other than medicinal values were significantly greater than those without any added values. A significant difference was also observed between the reported abundance and presence of added values of the ethnomedicinal plant species. The majority of ethnomedicinal plant species were used for firewood (25 species, 26%), forage (21 species, 22%), construction (14 species, 14%), food and fencing (3 species each, 3%), as well as timber, toothbrush and live fencing (2 species each, 2%) by the local people. However, many species (22 species, 23%) were indicated to have no added values other than their medicinal use (Figure 6).

## Discussion and conclusion

A remarkable traditional medicinal plant knowledge and practice was documented from the study area. Older traditional healers had greater knowledge and use of ethnomedicinal plant species than younger traditional healers. This may indicate that the indigenous medicinal plant use knowledge was declining among the younger generation, which could be attributed to the low interest of the younger generation to inherit and use ethnomedicinal knowledge. Another study by [23] also showed that medicinal plant knowledge and use increased with age when the community suffered an important erosion of ethnomedicinal plant knowledge.

The indigenous medicinal plant knowledge and use was independent of the educational level of traditional healers. This suggests that traditional healers could inherit the knowledge and use of ethnomedicine from parents as long as they belong to a knowledgeable family member irrespective of their educational status. This finding is in agreement with the work of [24] who reported that the proportion of healers who transferred their knowledge and those who did not was similar irrespective of their educational level. Results also revealed that many of the traditional healers reported to transfer their knowledge and use of ethnomedicinal plants orally to their favorite family member. Such transfer of indigenous knowledge is liable to erosion as it could vanish when knowledgeable elders die before the knowledge is transferred or during resettlements of individuals or communities [25,26].

The agreement among the traditional healers on the use of ethnomedicinal plant species was high for tumor, rabies, insect bite, rheumatism and *Rissaa*. This may indicate that the incidence of such diseases was relatively high in the study area. But consensus among the traditional healers was not observed for the majority of the diseases reported, which might be due to individual differences in the indigenous knowledge or the diverse backgrounds of healers [27].

High fidelity level was recoded on the use of *G. superba*, *R. communis, C. arereh, J. schimperiana and C. uncinulata *by traditional healers to treat tumor, rabies, insect bite, rheumatism and *Rissaa*, respectively. Low fidelity level for some of the species used against the aforementioned ailments shows that the species were used by the healers to treat many diseases.

The two species frequently used by traditional healers to treat tumor were *G. superba *and *P. zeylanica. P. zeylanica *was indicated to have antioxidant [28], antiviral [29], antiplasmodial [30], antibacterial [31,32] and stimulatory [33] properties. Even though these activities of *P. zeylanica *were not related to the traditional use in treating tumor, they may have therapeutic potential to treat rheumatism.

Anti-inflammatory activity from the methanolic extract of the roots of *R. communis *was reported by [34] while [35] reported a hepatoprotective effect of N-demethyl ricinine isolated from the leaves of this species. Anticonceptive effect [36,37] and antidote properties against scorpion venom [38] were also reported for this species. Moreover, [39] found the lectins from *R. communis *to inhibit HIV-1 reverse transcriptase. The latter antiviral activity of *R. communis *could validate its traditional use in the study area to treat rabies.

The species with the second highest fidelity level next to *J. schimperiana *used by healers to treat rheumatism was *C. macrostachyus*. Purgative and inflammatory activities were reported from seeds of this species [40]. *M. foetida*, which was found to have antiplasmodial activity [41] and *C. anisata*, which was screened to have antimicrobial [42,43] and hypoglycaemic [44] activities, were also used by traditional healers to manage rheumatism.

High diversity of ethnomedicinal plant species was reported by traditional healers. The diversity of ethnomedicinal plant species decreased with increasing altitude. A study conducted by [45] in New Zealand also showed that altitude had by far the strongest effect on species richness.

Leaves were the most reported plant parts in the preparation of remedies. The preference of leaves to other plant parts could be due to ease of preparation, preparation of medicinal teas [46] and the presence of more bioactive ingredients in leaves developed in response to phytophagous organisms since they are the most vulnerable parts of a plant [47]. The use of more than one medicinal plant species was reported by healers to treat health problems, which could be attributed to the additive or synergistic effects of the mixtures [48].

Preservation of remedies was not reported by healers of the study area since the remedies were used mainly in their fresh forms. This might also be attributed to the availability of ethnomedicinal plant species in the area as most of them were woody species. In North Peru, [49] reported that some remedies were prepared using dried plant material when fresh material was not available, and when the plant material had to be transported from other regions.

Traditional healers reported that they prescribed different doses of remedies for different age groups. Preferably, more amounts of remedies were given for adults than children to treat the same disease. Though such prescription differences were practiced, still the amount prescribed by healers for both children and adults might not confirm with the standard prescriptions in modern medical literature [50].

Though the majority of healers reported that the remedies used to treat ailments had no adverse effects on patients, few healers noted the presence of some side effects in some remedies prepared from certain species of ethnomedicinal plants. A similar study conducted by [24] also showed that most of the remedies reported by healers had no serious adverse effects except vomiting and temporary inflammations. However, the low recognition of adverse effects by traditional healers for the majority of remedy preparations coupled with the absence of antidotes for those remedies, even with reported adverse effects, might sometimes worsen the health problem of patients.

Most of the ethnomedicinal plant species used by healers to treat human ailments were reported to be rare and the abundance of ethnomedicinal plant species differed significantly with respect to the plant part used, plant condition used, marketability and multiple use of the species. This might be due to impact of such factors on anthropogenic pressure and the survival of ethnomedicinal plant species.

The findings of the current study showed that ethnomedicinal plants were under serious threat mainly due to deforestation and drought. Another study conducted by [51] at Bale Mountains National Park, Southeastern Ethiopia, also showed that deforestation for various purposes and agricultural expansion and intensification were the principal threats to medicinal plant species. A study by [52] also indicated that over-exploitation and deforestation were the main causes for the depletion of medicinal plants in northwest Yunnan, China. Although the medicinal plant species were under threat, traditional healers do not practice any conservation measures to ensure the sustainability of such plant resources. Therefore, interventions are required to mitigate the underlying threats of ethnomedicinal plant resources and ensure their conservation and sustainable utilization.

## Competing interests

The authors declare that they have no competing interests.

## Authors' contributions

HY and DY conceptualized and designed the study, collected field data, carried out statistical analysis and drafted the manuscript. DT participated in the data analysis and drafting as well as enrichment of the manuscript. All authors took part in approving the final manuscript.

## Supplementary Material

Additional file 1 The additional file contains pictures showing partial view of Gilgel Gibe Hydropower Reservoir area and some of the events during ethnobotanical data collection like interviews and field data collection. Click here for file

Additional file 2 Plant species of medicinal use to treat human ailments, parts used, mode of preparation and application. The additional file lists human ailments treated, scientific name of plant species used, family, local name, voucher number, part used, methods of preparation and application, and condition of part used. Click here for file
