# Supplementary material for: Ethnomedicinal plant knowledge and practice of the Oromo ethnic group in southwestern Ethiopia
Source: J Ethnobiol Ethnomed. 2008 Apr 29;4:11. doi: 10.1186/1746-4269-4-11 (PMC2390512; doi:10.1186/1746-4269-4-11)
Supplement: Additional file 1 — The additional file contains pictures showing partial view of Gilgel Gibe Hydropower Reservoir area and some of the events during ethnobotanical data collection like interviews and field data collection. [file 1746-4269-4-11-S1.ppt]

## Slide 1
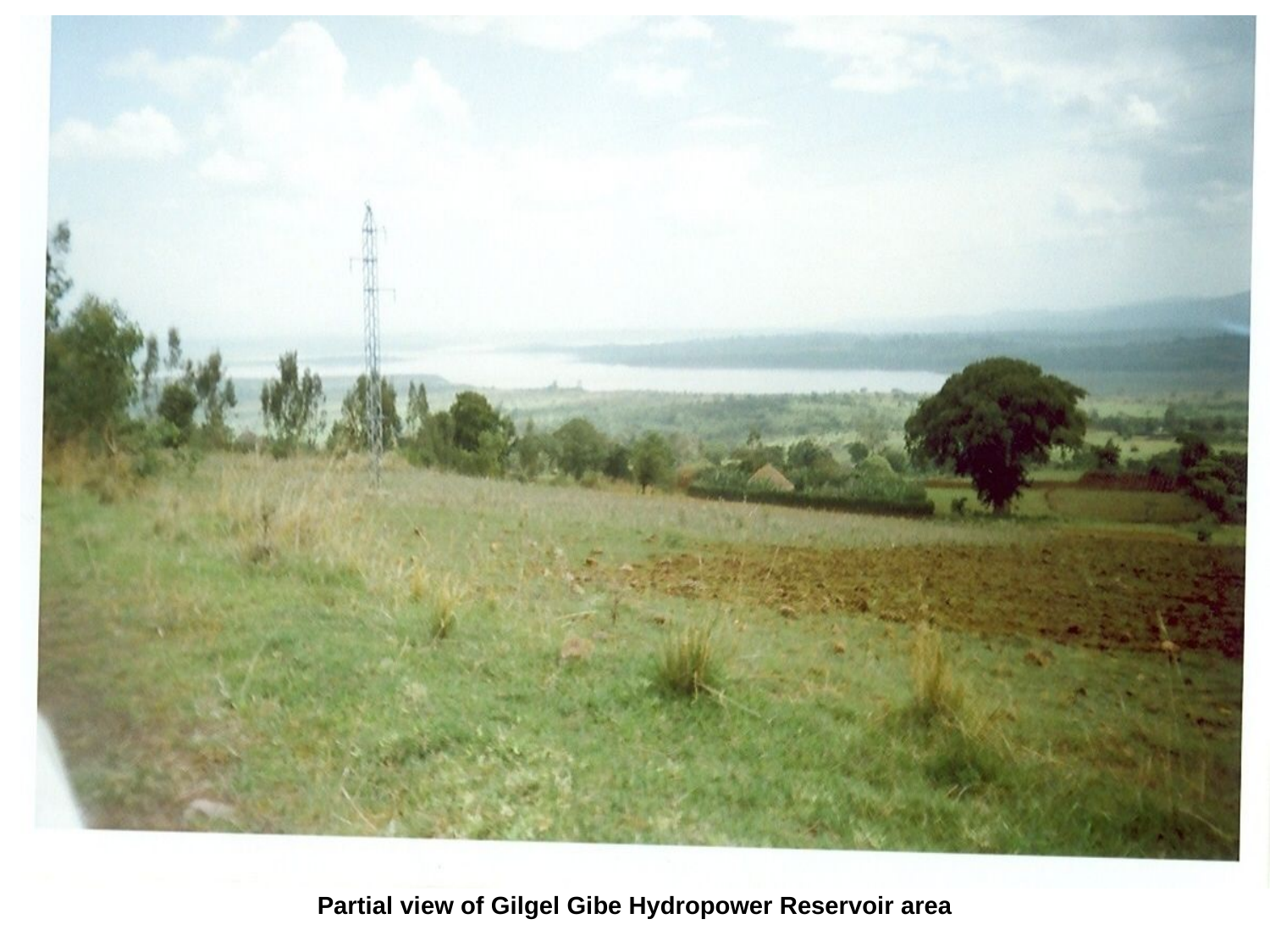

# Partial view of Gilgel Gibe Hydropower Reservoir area

## Slide 2
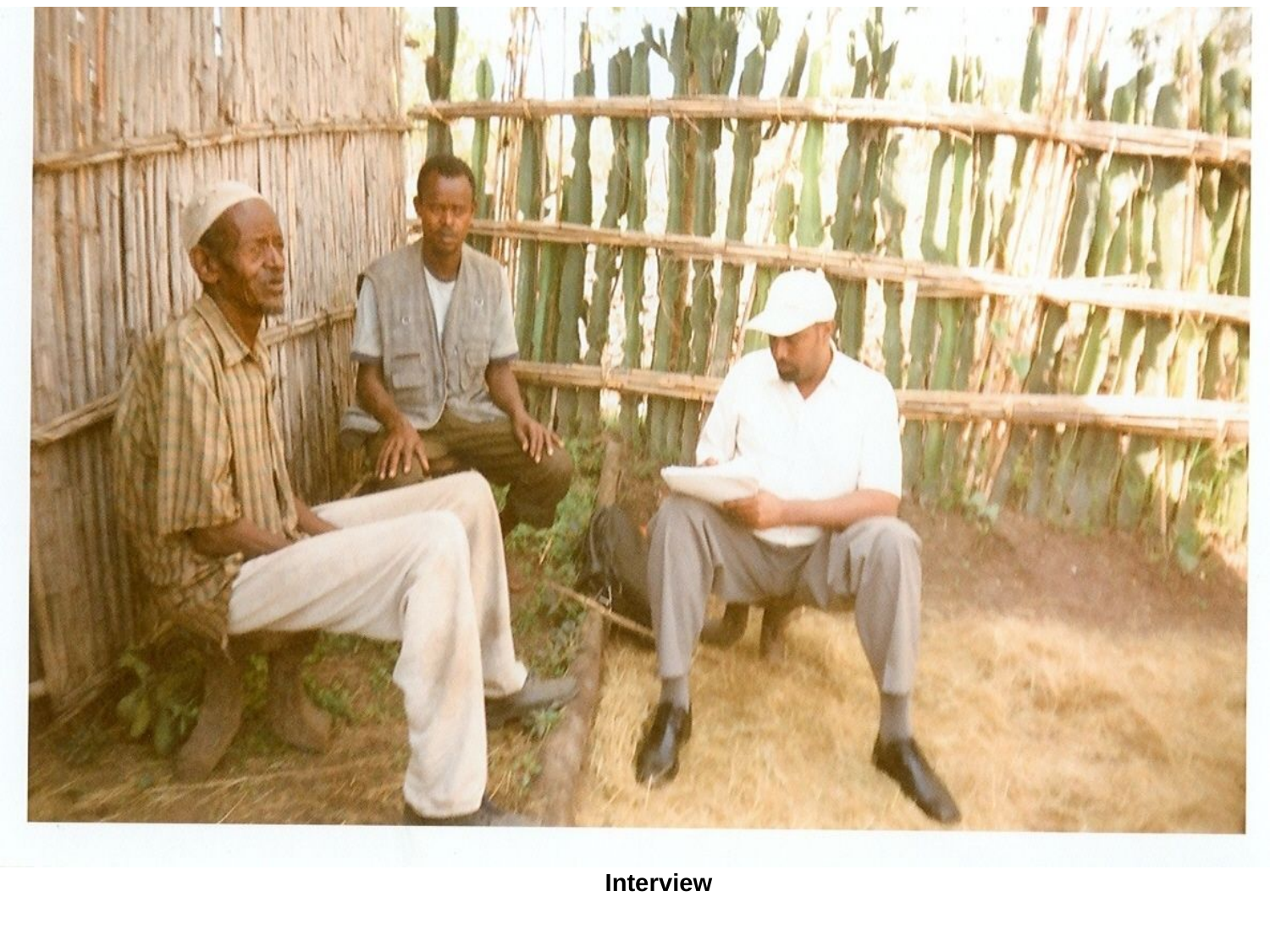

# Interview

## Slide 3
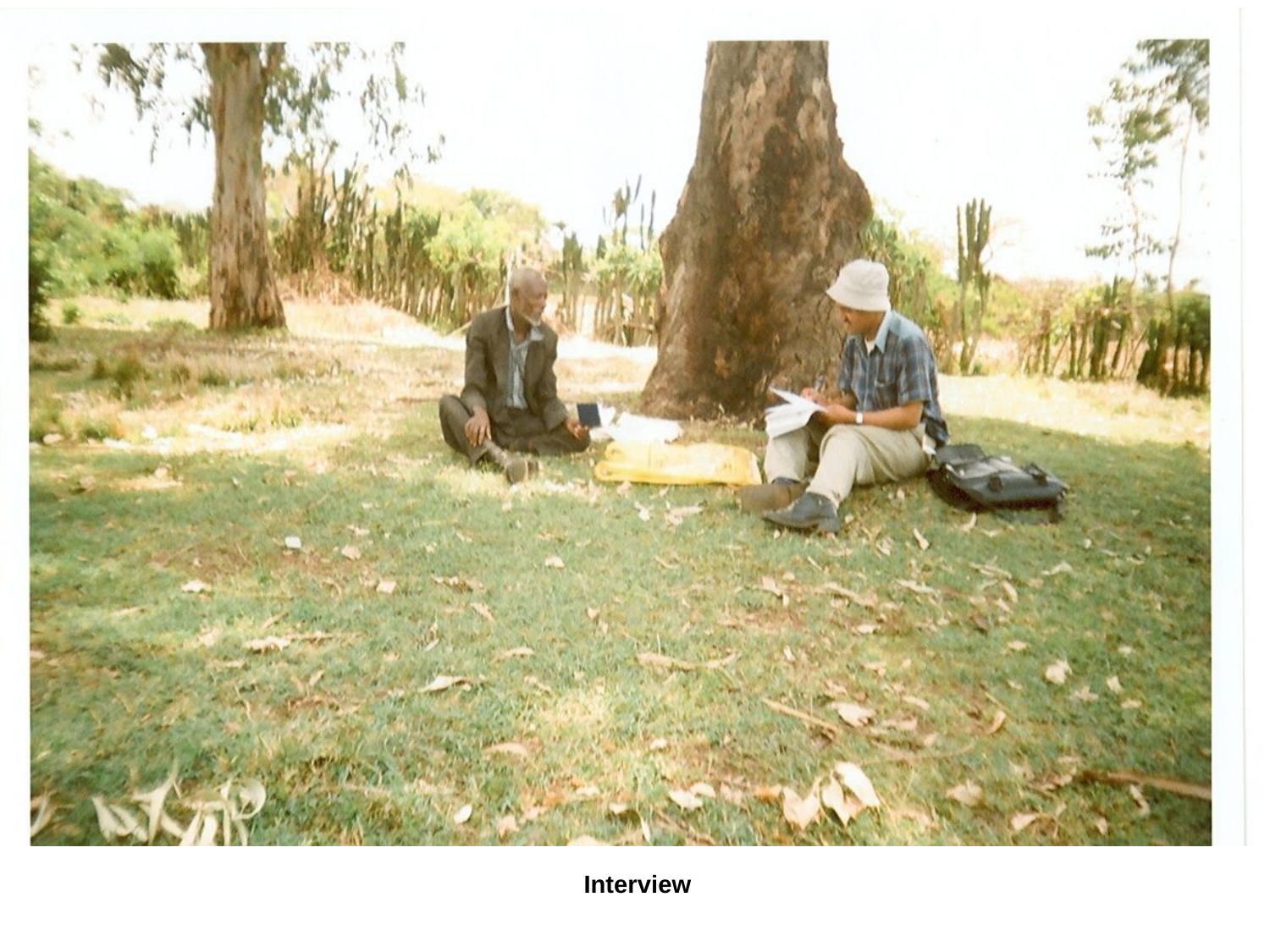

# Interview

## Slide 4
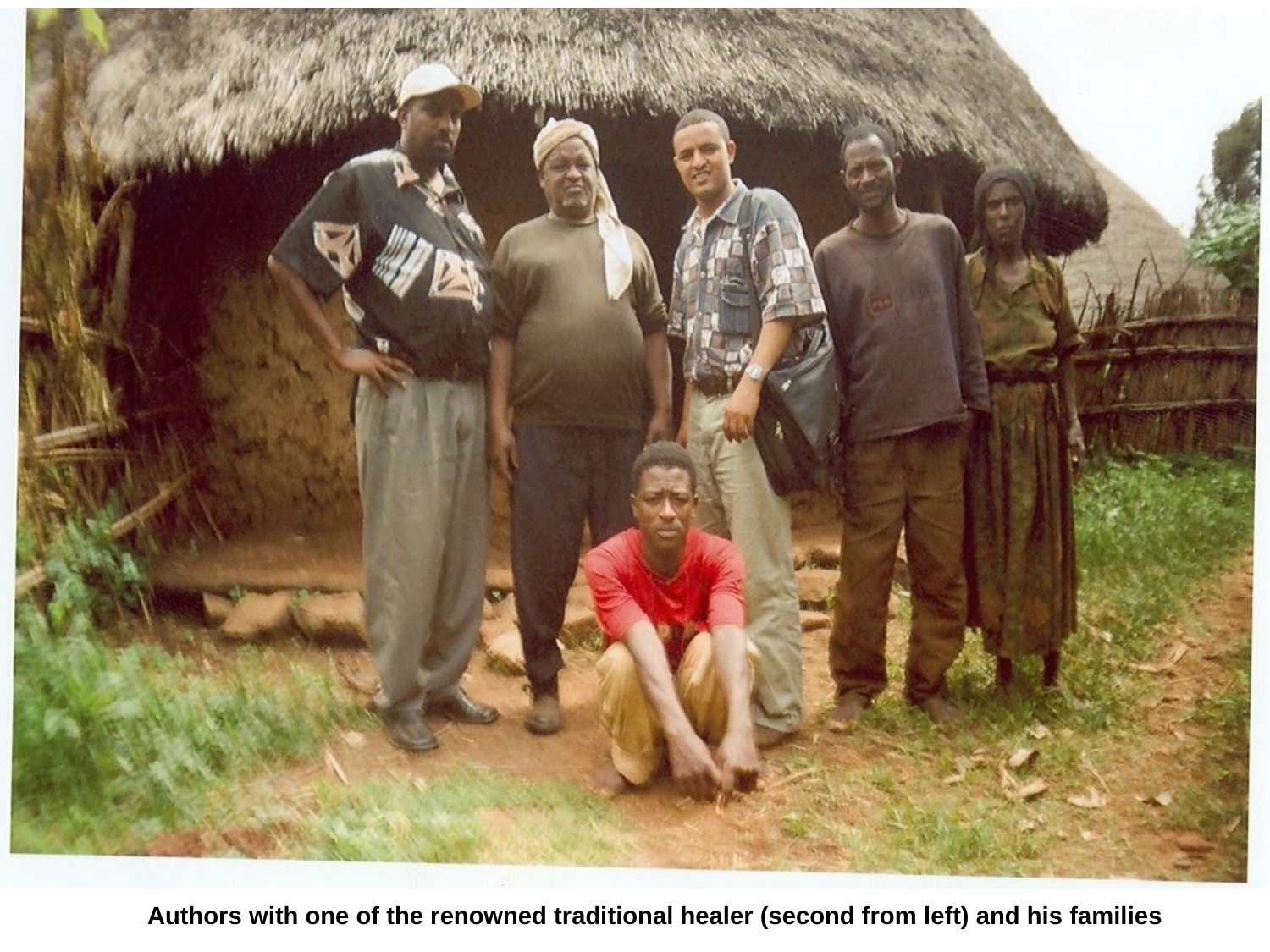

# Authors with one of the renowned traditional healer (second from left) and his families

## Slide 5
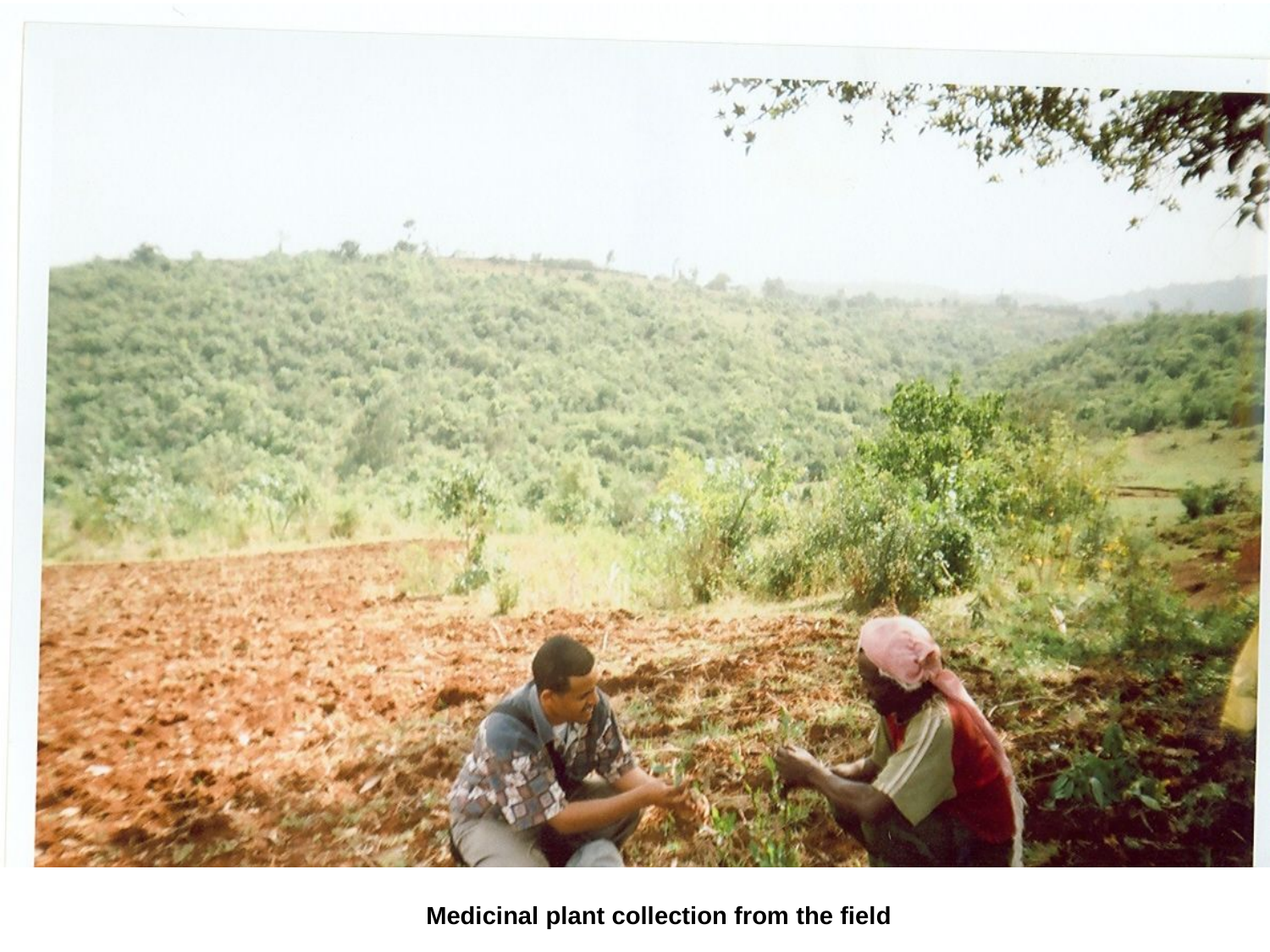

# Medicinal plant collection from the field

## Slide 6
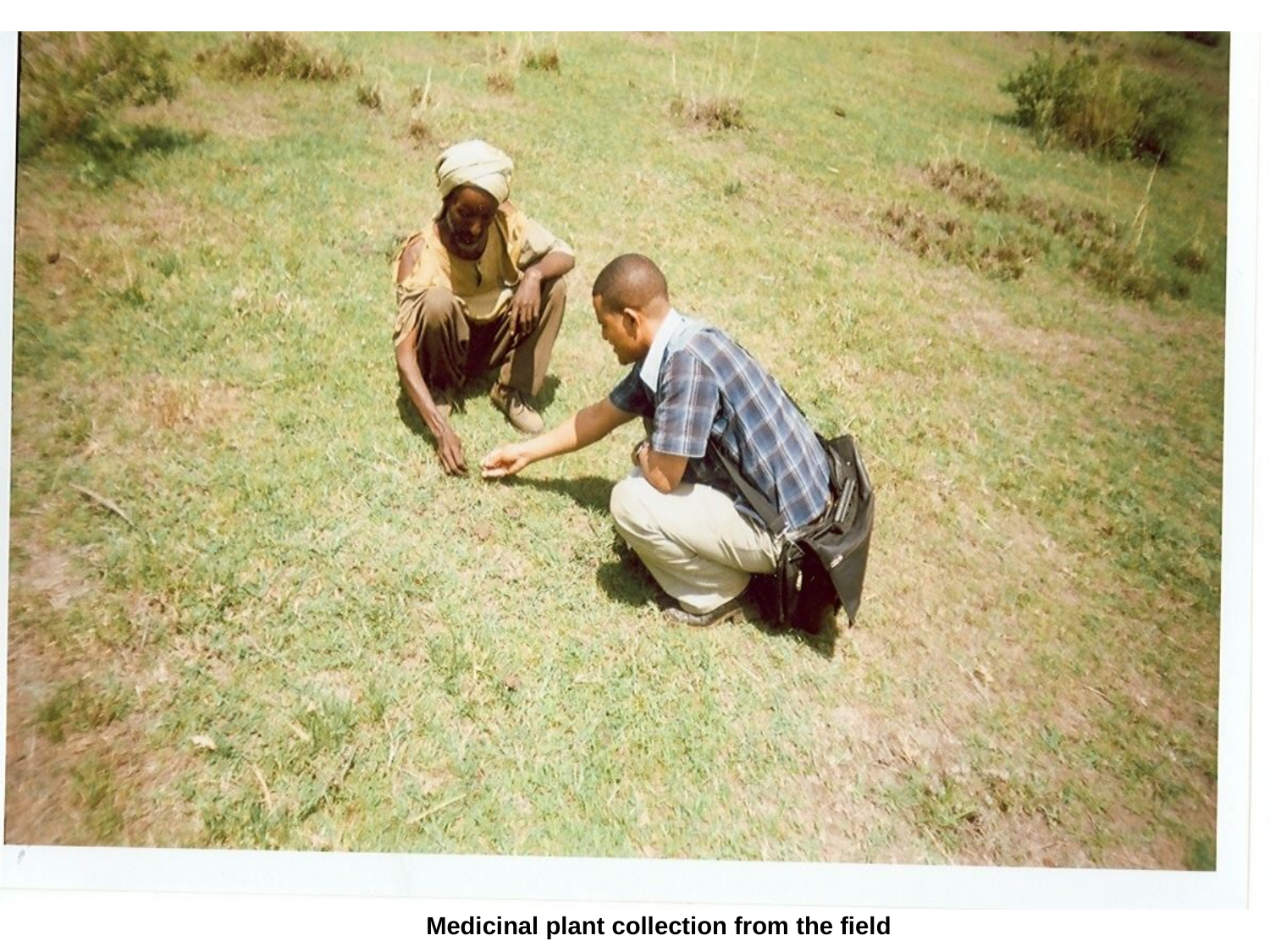

# Medicinal plant collection from the field
