# Supplementary material for: Ethnomedicinal plant knowledge and practice of the Oromo ethnic group in southwestern Ethiopia
Source: J Ethnobiol Ethnomed. 2008 Apr 29;4:11. doi: 10.1186/1746-4269-4-11 (PMC2390512; doi:10.1186/1746-4269-4-11)
Supplement: Additional file 2 — Plant species of medicinal use to treat human ailments, parts used, mode of preparation and application. The additional file lists human ailments treated, scientific name of plant species used, family, local name, voucher number, part used, methods of preparation and application, and condition of part used. [file 1746-4269-4-11-S2.pdf]

Additional file 2. Plant species of medicinal use to treat human ailments, parts used, mode of preparation and application

Human Disease	Scientific name	Family	Local name	Voucher	Part Used	Methods of preparation	Route of administration	Methods of application	Condition of part used
Abdominal distension (*Gara Kassa*)	*Momordica foetida* Schumach.	Cucurbitaceae	Hidda Boffaa/Buqee	Ha & De 48	Leaves	Crushed, squeezed, mixed with water	Oral	Drinking	Fresh
Afura lafaa (yhitsanatin fit yemiasabit)	*Cordia africana* Lam.	Boraginaceae	Wodessaa	Ha & De 85	Leaves	Extracted with cold water	Oral	Drinking	Fresh
	Datura stramonium L.	Solanaceae	Asangiraa	Ha & De 38	Leaves	Extracted with cold water	Oral	Drinking	Fresh
	Premna schimperi Engl.	Verbenaceae	Urgessa	Ha & De 4	Leaves	Extracted with cold water	Oral	Drinking	Fresh
	Clerodendrum myricoides (Hochst.) Vatke	Lamiaceae	Marasisa	Ha & De 82	Leaves	Extracted with cold water	Oral	Drinking	Fresh
Allergies (*Abiato, shererit*)	*Alysicarpus quartinianus* A. Rich.	Fabaceae	Hadhaa/Korcha Hadhaa	Ha & De 15	Root, leaves	Crushed, used alone	Topical	Topical application	Fresh
	Croton macrostachyus Del.	Euphorbiaceae	Makanissaa	Ha & De 6	Leaves	Pounded, squeezed; powdered, concocted	Topical	Topical application	Fresh or dried
	Vernonia amygdalina Del.	Asteraceae	Ebichaa	Ha & De 30	Leaves	Pounded, squeezed; powdered, concocted	Topical	Topical application	Fresh or dried
	Premna schimperi Engl.	Verbenaceae	Urgessa	Ha & De 4	Leaves	Pounded, squeezed; powdered, concocted	Topical	Topical application	Fresh or dried
Allergies (*Araabuu)*	*Oxalis radicosa* A. Rich.	Oxalidaceae	Soqideretii	Ha & De 21	Leaves	Squeezed with water	Oral	Drinking	Fresh
Allergies (*Ramoo*)	*Galium simense* Fresen.	Rubiaceae	Matane	Ha & De 94	Whole	Crushed, extracted with cold water	Topical	Topical application	Fresh
Amoebiasis	*Alysicarpus quartinianus* A. Rich.	Fabaceae	Hadhaa/Korcha Hadhaa	Ha & De 15	Root	Powdering, mixed with garlic	Oral	Swallowing	Dried

Additional file 2 (Continued)

Human Disease	Scientific name	Family	Local name	Voucher	Part Used	Methods of preparation	Route of administration	Methods of application	Condition of part used
Anaemia (*Dhidhina Mataa*)	*Solanecio angulatus* (Vahl) C. Jeffrey	Asteraceae	haqarqaraa/ gabisa	Ha & De 12	Leaves	Crushed, mixed with butter	Topical	Topical application	Fresh
Anaemia (*Hiireenaa Daga*)	*Hypoestes triflora* (Forssk.) Roem. & Schult.	Acanthaceae	Togoo	Ha & De 9	Leaves	Decoction, mixed with sugar	Oral	Drinking	Fresh
Asabal	*Chamaecrista mimosoides* (L.) Greene	Fabaceae	Qoricha Sabal	HA & De 56	Roots, fruits	Crushed, Squeezed with water	Oral	Drinking	Fresh
Blister (*Shao*)	*Solanum incanum* L.	Solanaceae	Hiddii	HA & De 16	Leaves & fruits	Crushed, squeezed, used alone	Topical	Ointment	Fresh
Constipation (*Bokokkaa*)	*Solanecio angulatus* (Vahl) C. Jeffrey	Asteraceae	haqarqaraa/ gabisa	Ha & De 12	Leaves	Squeezed with water	Oral	Drinking	Fresh
Cut (*Madaa*)	*Croton macrostachyus* Del.	Euphorbiaceae	Makanissaa	Ha & De 6	Leaves, leaf exudate	Crushed, used alone	Topical	Topical application	Fresh
Diabetes (*Dhukuba Lafaa*)	*Datura stramonium* L.	Solanaceae	Asangiraa	Ha & De 38	Leaves	Crushed, squeezed with water	Oral	Drinking	Fresh
	Sapium ellipticum (Krauss) Pax	Euphorbiaceae	Bosoqaa	Ha & De 57	Leaves	Crushed, squeezed with water	Oral	Drinking	Fresh
Diarrhoea (*Gara Kassaa*)	*Tragia mitis* Muell. Arg.	Euphorbiaceae	Guubduu	Ha & De 10	Root	Crushed, mixed with water and sugar	Oral	Drinking	Fresh
Diarrhoea (*Kaassaa*)	*Hypericum quartinianum* A. Rich.	Hypericaceae	Uleefoonii	Ha & De 82	Leaves	Extracted with cold water	Oral	Drinking	Fresh
	Hibiscus ovalifolius (Forssk.) Vahl	Malvaceae	Code 4	Ha & De 83	Leaves	Extracted with cold water	Oral	Drinking	Fresh
	Cyphostemma dembianense (Chiov.) Vollesen	Vitaceae	Code 5	Ha & De 23	Leaves	Extracted with cold water	Oral	Drinking	Fresh
Earache (*Tessissaa Gura*)	*Datura stramonium* L.	Solanaceae	Asangiraa	Ha & De 38	Seeds	Powdered, mixed with water	Auricular	Dropping	Dried
Eczema (*Chiefee, Abiatoo*)	*Ficus sycomorus* L.	Moraceae	Balantai/Balansofii	Ha & De 14	Stem bark	Powdering	Topical	Topical application	Dried
	Vernonia myriantha Hook.f.	Asteraceae	Reji	Ha & De 35	Leaves	Crushing	Topical	Topical application	Fresh

Additional file 2 (Continued)

**Human Disease**	**Scientific name**	**Family**	**Local name**	**Voucher**	**Part Used**	**Methods of preparation**	**Route of administration**	**Methods of application**	**Condition of part used**
Eczema (*Sibijii*)	*Sida ovata* Forssk.	Malvaceae	Karaabaa	Ha & De 87	Leaves	Crushed, used alone	Topical	Topical application	Fresh
	Erythrina brucei Schwinef.	Fabaceae	Wolensuu	Ha & De 20	Stem bark	Crushed, mixed with water	Topical	Washing, the lesions)	Fresh
	Thalictrum rhynchocarpum Dill. & A. Rich.	Ranunculaceae	Qoricha Sibijii	Ha & De 58	Roots	Powdered, mixed with water	Topical	Topical application	Dried
Evil eye (*Buda*)	*Withania somnifera* (L.) Dun.	Solanaceae	Qorcha Buda (gizawa)	Ha & De 41	Leaves	Squeezed, used alone	Oral	Drinking	Fresh
Febrile Illness (*Michii*)	*Ocimum lamiifolium* Hochst. ex Benth.	Lamiaceae	Damakessie	Ha & De 8	Leaves	Pounded, squeezed, used alone	Oral, Topical	Drinking, Rubbing	Fresh
Flu (*Busaa*)	*Ocimum lamiifolium* Hochst. ex Benth.	Lamiaceae	Damakessie	Ha & De 8	Leaves	Crushed, mixed with water	Oral	Drinking	Fresh
Gastritis (*Cheguara*)	*Clausena anisata* (Willd.) Benth.	Rutaceae	Ulmaye	Ha & De 37	Leaves	Crushed, decoction	Oral	Drinking	Fresh
Gland TB (*Naqarsaa*)	*Premna schimperi* Engl.	Verbenaceae	Urgessa	Ha & De 4	Leaves	Crushed, Squeezed with water	Oral	Drinking	Fresh
	Myrica salicifolia A. Rich.	Myricaceae	Nolee	Ha & De 59	Leaves	Crushed, Squeezed with water	Oral	Drinking	Fresh
	Myrsine africana L.	Myrsinaceae	Qachamaa	Ha & De 60	Leaves	Crushed, Squeezed with water	Oral	Drinking	Fresh
	Clausena anisata (Willd.) Benth.	Rutaceae	Ulmaye	Ha & De 37	Leaves	Crushed, Squeezed with water	Oral	Drinking	Fresh
	Alysicarpus quartinianus A. Rich.	Fabaceae	Hadhaa/Korcha Hadhaa	Ha & De 15	Leaves	Crushed, Squeezed with water	Oral	Drinking	Fresh
Gonnorrhoea (*Dhukuba Dhiraa, Chebto)*	*Momordica foetida* Schumach.	Cucurbitaceae	Hidda Boffaa/Buqee	Ha & De 48	Roots	Crushed, squeezed with water	Intravenous	Injection	Fresh
	Croton macrostachyus Del.	Euphorbiaceae	Makanissaa	Ha & De 6	Fruits, root	Pounded, mixed with water	Oral	Drinking	Fresh
	Kanahia laniflora (Forssk.) R. Br.	Asclepiadaceae	Shershera	Ha & De 64	Root	Pounded, mixed with water	Oral	Drinking	Fresh

Additional file 2 (Continued)

Human Disease	Scientific name	Family	Local name	Voucher	Part Used	Methods of preparation	Route of administration	Methods of application	Condition of part used
Gonnorrhoea (*Dhukuba Dhiraa, Chebto)*	*Oreosyce africana* Hook.f.	Cucurbitaceae	Hiddii	Ha & De 45	Leaves	Concoction, Crushed, squezed with water	Oral	Drinking	Fresh
	Entada abyssinica Steud. ex A. Rich.	Fabaceae	Haambaltaa	Ha & De 46	Leaves	Concoction, Crushed, squezed with water	Oral	Drinking	Fresh
Haemorrhoids (*Kormomo, Kintaroti*)	*Gardenia ternifolia* Schumach. & Thonn.	Rubiaceae	Kambeelloo/Gambe elloo	Ha & De 5	Fruit	Crushed, powdered, concoction, used alone	Topical	Topical application	Fresh or dried
Headache (*Bowo Mata*)	*Vernonia amygdalina* Del.	Asteraceae	Ebichaa	Ha & De 30	Leaves	Pounded, mixed with water	Oral	Drinking	Fresh
Headache (*Bowo*)	*Ocimum lamiifolium* Hochst. ex Benth.	Lamiaceae	Damakessie	Ha & De 8	Leaves	Squeezed with water	Nasal	Dropping	Fresh
Heart failure (*Yelib dikam*)	*Entada abyssinica* Steud. ex A. Rich.	Fabaceae	Haambaltaa	Ha & De 46	Stem bark, leaves	Crushed, powdered, used alone	Oral	Drinking	Dried
Helimenthic infection (*Raamoo*)	*Citrus limon* (L.) Burm. f.	Rutaceae	Lomiya	Ha & De 13	Fruit	Squeezed alone, mixed with water	Oral	Drinking	Fresh
Insect bite (*Hadhaa*)	*Alysicarpus quartinianus* A. Rich.	Fabaceae	Hadhaa/Korcha Hadhaa	Ha & De 15	Leaves	Crushed, warmed, used alone	Topical	Topical application	Fresh
	Cassia arereh Del.	Fabaceae	Botoroo	Ha & De 18	Stem bark	Crushed, used alone	Topical	Topical application	Fresh or dried
	Alysicarpus quartinianus A. Rich.	Fabaceae	Hadhaa/Korcha Hadhaa	Ha & De 15	Root	Crushed, used alone	Topical	Topical application	Fresh
	Canavalia africana Dunn	Fabaceae	Otongoraa (adenguare)	Ha & De 92	Leaves	Crushed, warmed, used alone	Topical	Topical application	Fresh
Loss of hair (*Toro*)	*Afrocarpus falcatus* (Thunb.) C. N. Page	Podocarpaceae	Birbirsa	Ha & De 107	Leaves	Crushed, mixed with butter	Topical	Topical application	Fresh
	Solanum incanum L.	Solanaceae	Hiddii	HA & De 16	Leaves	Crushed, mixed with butter	Topical	Topical application	Fresh
Madness (*Maraatuu)*	*Salix subserrata* Willd.	Salicaceae	Aleltu	HA & De 53	Leaves	Concoction, crushed, squeezed with water	Oral	Drinking	Fresh

Additional file 2 (Continued)

Human Disease	Scientific name	Family	Local name	Voucher	Part Used	Methods of preparation	Route of administration	Methods of application	Condition of part used
Madness (*Maraatuu)*	*Datura stramonium* L.	Solanaceae	Asangiraa	Ha & De 38	Leaves	Concoction, crushed, squeezed with water	Oral	Drinking	Fresh
	Justicia schimperiana (Hochst. ex Nees) T. Anders.	Acanthaceae	Dhumugaa	Ha & De 26	Leaves	Concoction, crushed, squeezed with water	Oral	Drinking	Fresh
	Clerodendrum myricoides (Hochst.) Vatke	Lamiaceae	Marasisa	Ha & De 82	Leaves	Concoction, crushed, squeezed with water	Oral	Drinking	Fresh
Malaria (*Bossa*)	*Gardenia ternifolia* Schumach. & Thonn.	Rubiaceae	Kambeelloo/Gambe elloo	Ha & De 5	Stem bark	Crushed, extracted with cold water	Oral	Drinking	Dried
Menustrual disorder (yalememtat)	*Gloriosa superba* L.	Colchicaceae	Dawaurahman/Rah maldawa	Ha & De 3	Roots	Crushed, mixed with sugar	Oral	Swallowing	Fresh
Nasal infection (*Afincha legema*)	*Carissa spinarum* L.	Apocynaceae	Hagamssaa	Ha & De 90	Root	enclosing a piece of the root in slice of close for smelling	Nasal	Smelling	Fresh
Nerve pbm (*Hananassaa, Gogosca*)	*Bridelia micrantha* (Hochst.) Baill.	Euphorbiaceae	Muka Chito (Muka Morma)	Ha & De 17	Leaves	Crushed, mixed with water	Topical	Washing/bathin g	Fresh
Nose bleeding (*Fununa*)	*Solanum incanum* L.	Solanaceae	Hiddii	HA & De 16	Leaves	Squeezed, used alone	Nasal	Dropping	Fresh
Ophthalmia (*Dhukuba Eja*)	*Bidens pilosa* L.	Asteraceae	Darbattaa	Ha & De 102	Leaves	Squeezing, concoction	Optical	Dropping	Fresh
	Hypoestes triflora (Forssk.) Roem. & Schult.	Acanthaceae	Togoo	Ha & De 9	Leaves	Squeezed with water	Optical	Topical application	Fresh
	Calpurnia aurea (Ait.) Benth.	Fabaceae	Ceekaa	Ha & De 39	Leaves	Squeezing, concoction	Optical	Dropping	Fresh
Otorrhoea (*Dhukuba Guraa*)	*Plectranthus rupestris* Vatke ex Baker	Lamiaceae	Balajuu	Ha & De 47	Leaves	Crushed, squeezed with water	Auricular	Dropping	Fresh
	Clematis hirsuta Perr. & Guill.	Ranunculaceae	Fitii	Ha & De 99	Leaves	Crushed, squeezed with water	Auricular	Dropping	Fresh

Additional file 2 (Continued)

Human Disease	Scientific name	Family	Local name	Voucher	Part Used	Methods of preparation	Route of administration	Methods of application	Condition of part used
Otorrhoea (*Dhukuba Guraa*)	*Myrica salicifolia* A. Rich.	Myricaceae	Nolee	Ha & De 59	Leaves	Crushed, squeezed with water	Auricular	Dropping	Fresh
	Calpurnia aurea (Ait.) Benth.	Fabaceae	Ceekaa	Ha & De 39	Leaves	Crushed, squeezed with water	Auricular	Dropping	Fresh
	Englerina woodfordioides (Schweinf.) M. Gilbert	Loranthaceae	Harmee	Ha & De 51	Leaves	Crushed, squeezed with water	Auricular	Dropping	Fresh
Otorrhoea (*Guraa Maluu*)	*Crotalaria spinosa* Hochst. ex Benth.	Fabaceae	Code A	Ha & De 77	Root	Pounded, powdered, mixed with water	Auricular	Dropping	Dried
Oversweating and fever (*Lelabna lemuqet*)	*Justicia schimperiana* (Hochst. ex Nees) T. Anders.	Acanthaceae	Dhumugaa	Ha & De 26	Leaves	Squeezing, used alone	Oral	Drinking	Fresh
Poisoning (*Merz lebela sew*)	*Datura stramonium* L.	Solanaceae	Asangiraa	Ha & De 38	Leaves (apical meristem)	Crushed, squeezed, used alone	Oral	Drinking	Fresh
Rabies (*Dhukuba seree*)	*Salix subserrata* Willd.	Salicaceae	Aleltu	HA & De 53	Leaves	Crushed, mixed with water & milk	Oral	Drinking	Fresh
	Afrocarpus falcatus (Thunb.) C. N. Page	Podocarpaceae	Birbirsa	Ha & De 107	Leaves	Crushed, mixed with water & milk	Oral	Drinking	Fresh
Rabies (*Labedewusha*)	*Ricinus communis* L.	Euphorbiaceae	Koboo	Ha & De 40	Leaves	Pounding, squeezed, mixed with milk	Oral	Drinking	Fresh
Rabies (*Wusha lenekesew sew*)	*Ricinus communis* L.	Euphorbiaceae	Koboo	Ha & De 40	Leaves	Extracted with tea	Oral	Drinking	Fresh
Rheumatism (*Gnaataa*)	*Dregea schimperi* (Decne.) Bullock	Asclepiadaceae	Hida Adii/Haayotee	Ha & De 25	Leaves	Squeezed with water	Topical	Washing	Fresh
	Plumbago zeylanica L.	Plumbaginaceae	Umeeraa	Ha & De 11	Root	Powdered, decoction	Oral	Drinking	Dried
	Clutia abyssinica Jaub. & Spach.	Euphorbiaceae	Tasuu	Ha & De 74	Leaves	Crushed, mixed with water	Oral, Topical	Drinking, washing	Fresh
	Momordica foetida Schumach.	Cucurbitaceae	Hidda Boffaa/Buqee	Ha & De 48	Leaves	Squeezed with water	Topical	Washing	Fresh
	Croton macrostachyus Del.	Euphorbiaceae	Makanissaa	Ha & De 6	Leaves	Squeezed with water	Topical	Washing	Fresh

Additional file 2 (Continued)

**Human Disease**	**Scientific name**	**Family**	**Local name**	**Voucher**	**Part Used**	**Methods of preparation**	**Route of administration**	**Methods of application**	**Condition of part used**
Rheumatism (*Gnaataa*)	*Justicia schimperiana* (Hochst. ex Nees) T. Anders.	Acanthaceae	Dhumugaa	Ha & De 26	Leaves	Squeezed with water	Topical	Washing	Fresh
	Ocimum gratissimum L.	Lamiaceae	Hancabii	Ha & De 93	Leaves	Squeezed with water	Topical	Washing	Fresh
	Calpurnia aurea (Ait.) Benth.	Fabaceae	Ceekaa	Ha & De 39	Leaves	Squeezed with water	Topical	Washing	Fresh
	Bersama abyssinica Fresen.	Melianthaceae	Lolchissaa	Ha & De 70	Leaves	Crushed, mixed with water	Oral, Topical	Drinking, washing	Fresh
	Clausena anisata (Willd.) Benth.	Rutaceae	Ulmaye	Ha & De 37	Leaves	Crushed, mixed with water	Oral, Topical	Drinking, washing	Fresh
	Justicia schimperiana (Hochst. ex Nees) T. Anders.	Acanthaceae	Dhumugaa	Ha & De 26	Leaves	Squeezed with water	Topical	Topical application	Fresh
	Croton macrostachyus Del.	Euphorbiaceae	Makanissaa	Ha & De 6	Leaves	Squeezed with water	Topical	Topical application	Fresh
	Celtis africana Burm.f.	Ulmaceae	Mataqoma (Muka Morma)	Ha & De 54	Leaves	Squeezed with water	Topical	Topical application	Fresh
Rheumatism (Qora)	*Plumbago zeylanica* L.	Plumbaginaceae	Umeeraa	Ha & De 11	Root	Powdered, mixed with water, honey, garlic, *Nigela sativa*	Oral	Swallowing	Dried
Rheumatism (*Sanba Naqarsa*)	*Croton macrostachyus* Del.	Euphorbiaceae	Makanissaa	Ha & De 6	Stem bark	Pounding, or powdering, mixed with water or 'aguat' or tea	Oral	Drinking	Fresh or dried
Ringworm & Tinea versicolor (*Chirtna quaqucha*)	*Olea europae* L. subsp. *cuspidata* (Wall. ex G.Don) Cif.	Oleaceae	Ejersa	Ha & De 76	Stem oil	Extracting oil by boiling stem	Topical	Ointment	Fresh
Rissaa	*Clerodendrum myricoides* (Hochst.) Vatke	Lamiaceae	Free	Ha & De 82	Root	Crushed, pounded, mixed with water	Oral	Drinking	Fresh
			Qitel	Ha & De 82	Root	Crushed, pounded, mixed with water	Oral	Drinking	Fresh

Additional file 2 (Continued)

Human Disease	Scientific name	Family	Local name	Voucher	Part Used	Methods of preparation	Route of administration	Methods of application	Condition of part used
Rissaa	*Desmodium repandum* (Vahl) DC.	Fabaceae	Shicha	Ha & De 67	Leaves	Crushed, used alone	Topical	Topical application	Fresh
	Croton macrostachyus Del.	Euphorbiaceae	Makanissaa	Ha & De 6	Leaves	Concoction, Crushed, mixed with water	Oral	Drinking	Fresh
	Cyathula uncinulata (Shrad.) Schinz	Amaranthaceae	Matane	Ha & De 29	Leaves	Concoction, Crushed, mixed with water	Oral	Drinking	Fresh
Scabies (*Ciitto*)	*Maesa lanceolata* Forssk.	Myrsinaceae	Eija Abbayi	Ha & De 43	Seeds	Powdering, mixed with edible oil	Topical	Topical application	Dried
	Englerina woodfordioides (Schweinf.) M. Gilbert	Loranthaceae	Harmee	Ha & De 51	Roots	Crushed, mixed with water & baseline	Topical	Ointment	Fresh
Skin crack (*Milla Dhodoi*)	*Alysicarpus quartinianus* A. Rich.	Fabaceae	Hadhaa/Korcha Hadhaa	Ha & De 15	Stem bark	Powdering, decoction	Oral	Drinking	Dried
Snake bite (*Cininee*)	*Cynodon dactylon* (L.) Pers.	Poaceae	Margaa (Chekorsa)	Ha & De 19	Leaves	Squeezed with water	Oral	Drinking	Fresh
	Cassia arereh Del.	Fabaceae	Botoroo	Ha & De 18	Stem bark	Powdering, mixed with tea	Oral	Drinking	Dried
Stabbing pain (*Woransa*)	*Stephania abyssinica* (Dillon & A. Rich.) Walp.	Menispermaceae	Kalala	Ha & De 72	Leaves	Powdering, used alone	Oral	Smoking	Dried
Stabbing pain (*wugat*)	*Carissa spinarum* L.	Apocynaceae	Hagamssaa	Ha & De 90	Leaves	Smoke bath	Smoke bath	Smoke bath	Fresh
Stomachache (*Garaa Kutataa*)	*Clerodendrum myricoides* (Hochst.) Vatke	Lamiaceae	Marasisa	Ha & De 82	Root	Extracted with cold water	Oral	Drinking	Fresh
	Plumbago zeylanica L.	Plumbaginaceae	Umeeraa	Ha & De 11	Root	Extracted with cold water	Oral	Drinking	Fresh
Stress (*Hiireenaa*)	*Solanecio angulatus* (Vahl) C. Jeffrey	Asteraceae	haqarqaraa/ gabisa	Ha & De 12	Leaves	Crushed, mixed with butter	Topical	Topical application	Fresh
Stress (*Ye aimiro chinqet*)	*Caesalpinia decapetala* (Roth) Alston	Fabaceae	Arangama Gurachaa	Ha & De 36	Stem bark	Powdered, wrapped with cotton clothe, used alone	Nasal	Sniffing	Dried
Tinea versicolor (*Sono*)	*Dioscorea praehensilis* Benth.	Dioscoreaceae	Wociinoo	Ha & De 31	Leaves	Crushed, used alone	Topical	Brushing (Topical application)	Fresh
Tonsilitis (*Huuba Qonqoo*)	*Flacourtia indica* (Burm. f.) Merr.	Flacourtiaceae	Akokoo	Ha & De 33	Leaves	used alone (No body contact)	No body contact	Spiritual	Dried

Additional file 2 (Continued)

**Human Disease**	**Scientific name**	**Family**	**Local name**	**Voucher**	**Part Used**	**Methods of preparation**	**Route of administration**	**Methods of application**	**Condition of part used**
Tonsilitis (*Huuba Qonqoo*)	*Acmella coulirhiza* Del.	Asteraceae	Gororsa	Ha & De 42	Flower	Crushed, used alone	Oral	Chewing and swallowing	Fresh
	Cynodon dactylon (L.) Pers.	Poaceae	Chekorsaa	Ha & De 19	Leaves	used alone (No body contact)	No body contact	Spiritual	Dried
Toothache (*Dhukuba Ilkanii*)	*Premna schimperi* Engl.	Verbenaceae	Urgessa	Ha & De 4	Leaves	Used alone	Dental	Chewing	Fresh
	Calpurnia aurea (Ait.) Benth.	Fabaceae	Ceekaa	Ha & De 39	Leaves	Crushed, used alone	Oral	Smoking	Dried
	Cyathula uncinulata (Shrad.) Schinz	Amaranthaceae	Metenaa	Ha & De 29	Root	Used alone	Dental	Chewing	Fresh
Tumor (*Tanachaa*)	*Tapinanthus globiferus* (A. Rich.) Tieghem	Loranthaceae	Dheertu Makanissaa	Ha & De 98	Leaves	Crushed, mixed with water	Oral	Drinking, Swallowing	Fresh
	Gloriosa superba L.	Colchicaceae	Dawaurahman/Rahmaldawa	Ha & De 3	Root	Powdering, given with tea & coffee	Oral	Drinking	Dried
	Plumbago zeylanica L.	Plumbaginaceae	Umeeraa	Ha & De 11	Root	Powdered, mixed with water and sugar	Oral	Drinking	Dried
	Plumbago zeylanica L.	Plumbaginaceae	Umeeraa	Ha & De 11	Twigs	Used alone	Put on the neck as necklace	Put on the neck as necklace	Fresh or dried
	Gloriosa superba L.	Colchicaceae	Dawaurahman/Rahmaldawa	Ha & De 3	Root	Crushed, mixed with water	Oral	Drinking	Fresh
Unable to urinate (*Dhukuba Garaa Itesuu)*	*Phytolacca dodecandra* L'Hérit.	Phytolaccaceae	Andodee	Ha & De 106	Root	Pounding, mixed with water and butter	Oral	Drinking	Fresh
Wound (*Qushuurii*)	*Sida ovata* Forssk.	Malvaceae	Karaabaa	Ha & De 87	Leaves	Crushed, used alone	Topical	Topical application	Fresh
